# Tracheobronchial mucoepidermoid carcinoma in four pediatric patients

**DOI:** 10.1016/j.rmcr.2026.102408

**Published:** 2026-03-20

**Authors:** Kalen Hendra, Muhammad Ijlal Khan, Mitali Thanawala, Manuel Silva-Carmona, Eric D. Zee

**Affiliations:** a Department of Pediatrics, Division of Pediatric Pulmonary Medicine, Stanford University School of Medicine, Palo Alto, CA, USA; b Department of Medicine, Division of Pulmonary, Critical Care and Sleep Medicine, University of Kentucky, Lexington, KY, USA; c Department of Pediatrics, Division of Pediatric Pulmonary Medicine, Baylor College of Medicine, Texas Children's Hospital, Houston, TX, USA

**Keywords:** Mucoepidermoid carcinoma, Tracheobronchial mucoepidermoid carcinoma, Pulmonary mucoepidermoid carcinoma, Pediatric, Lung neoplasm, Pediatric interventional pulmonology

## Abstract

Pediatric tracheobronchial mucoepidermoid carcinoma (MEC), also called pulmonary MEC, is a rare malignant primary lung neoplasm that is typically found in the large airways. Patients may present with symptoms such as cough, pneumonia, fever, hemoptysis, dyspnea, wheezing, chest pain, weight loss, and fatigue. These nonspecific symptoms (or lack of symptoms in some cases) often result in a delayed diagnosis. Chest CT typically demonstrates findings consistent with bronchial obstruction and diagnosis is made via histopathology. Surgical resection is the mainstay of treatment and children often have favorable outcomes. Here we present four cases of tracheobronchial MEC in children treated at three different hospitals. Our cases highlight the importance of having a broad differential that includes tracheobronchial MEC when a patient does not respond as expected to treatments such as inhaled steroids or antibiotics, and especially if the patient has hemoptysis and/or weight loss. As the use of pediatric interventional pulmonology becomes more common, we may see interventional pulmonology techniques utilized in the diagnosis and management of tracheobronchial MEC.

## Highlights

• Tracheobronchial MEC requires a high index of suspicion for a timely diagnosis
• Hemoptysis, weight loss, and respiratory symptoms warrant cross-sectional imaging
• Interventional pulmonology may be used to diagnose tracheobronchial MEC
• Interventional pulmonology may aid in the management of tracheobronchial MEC
• Further studies on the use of interventional pulmonology in pediatrics are needed

## 1 Introduction

Tracheobronchial mucoepidermoid carcinoma (MEC), also called pulmonary MEC, is a rare malignant primary lung neoplasm that accounts for 9-10% of all pediatric malignant primary lung neoplasms [1,2]. It is a salivary gland-type tumor that arises from mucous glands within the respiratory submucosa and is typically found in the large airways (trachea, carina, mainstem bronchi, and proximal lobar bronchi) [[1], [2], [3], [4], [5], [6], [7], [8]]. Patients may present with symptoms such as cough (most commonly), pneumonia, fever, hemoptysis, dyspnea, wheezing, chest pain, weight loss, and fatigue; however, some patients may be asymptomatic [1,4,6,7,[9], [10], [11], [12]]. Due to non-specific symptoms that are often attributed to other causes (such as infection or asthma) and the rarity of tracheobronchial MEC, patients often have a delayed diagnosis, with prior studies reporting a duration of 2-30 months between onset of symptoms and final diagnosis [[13], [14], [15], [16], [17]]. Chest computed tomography (CT) typically demonstrates findings consistent with bronchial obstruction such as pneumonia, atelectasis, air trapping, and bronchial dilatation with mucoid impaction. Tracheobronchial MECs may also have foci of calcification within the tumor itself that can be visualized on chest CT and histologic evaluation [18]. The diagnosis is made via histopathology, and the tumor is classified as low-grade, intermediate-grade, or high-grade. Tracheobronchial MEC is treated with complete surgical resection, and pediatric patients have good outcomes, with 96% disease-free survival at follow-up [4]. Here we report four cases of tracheobronchial MEC in children treated at three different hospitals.

## 2 Case presentations

### 2.1 Case 1

**Fig. 1:**
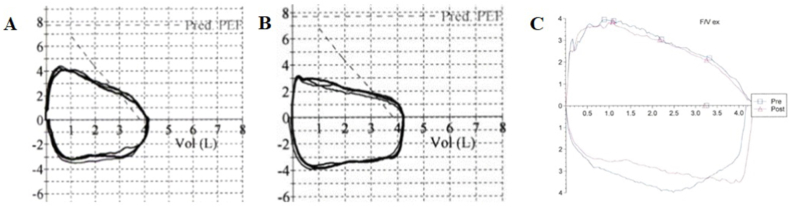
Spirometry for case 1. Spirometry performed at A) 9 months, B) 11 months, and C) 12 months after initial presentation to his pediatrician's office. Obstruction was noted in all three studies [forced expiratory volume in 1 second (FEV1)/forced vital capacity (FVC) ratios were 79%, 60%, and 73%, respectively]. Flattening of the expiratory loop was identified in the second study (B). Although flattening of the inspiratory loops is present in all three studies, it was not identified until after the tracheal mass was seen on bronchoscopy.

**Fig. 2:**
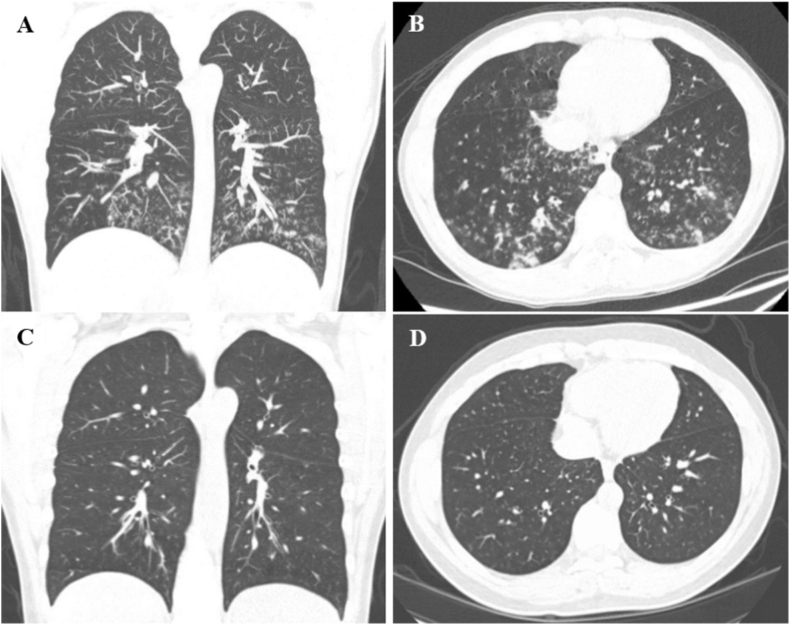
Chest CTs for case 1. A and B) Initial chest CT demonstrated innumerable patchy irregular pulmonary nodules throughout the mid and lower lungs bilaterally. C and D) Repeat chest CT 3 months after tumor debulking demonstrated resolution of the previously noted bilateral pulmonary nodules.

**Fig. 3:**
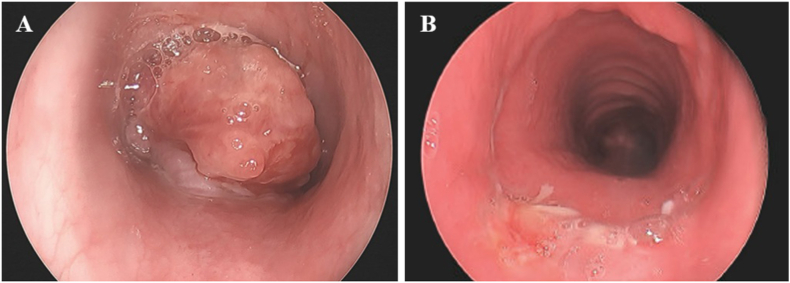
Bronchoscopies for case 1. A) Initial bronchoscopy revealed an endotracheal mass located 13 mm below the vocal cords with 90% occlusion of the trachea. B) Repeat bronchoscopy visualized the trachea 12 weeks after removal of the mass and 2 weeks after cricotracheal resection with tracheal anastomosis.

A previously healthy 14-year-old male presented with persistent cough, wheezing, and exercise intolerance that developed a month after a COVID-19 infection. He was diagnosed with asthma and treated with inhaled corticosteroids and a long-acting beta agonist (ICS-LABA) with some improvement in his symptoms. Serial spirometry demonstrated mild to moderate obstruction, though in hindsight there was persistent flattening of both the inspiratory and expiratory flow volume loops that was not previously identified (Fig. 1A, B and C). Chest x-rays were normal. His symptoms persisted and about a year after his initial presentation he was noted to have new rhonchi, crackles, and a prolonged expiratory phase and was started on airway clearance therapies. Upon further review, he had had two episodes of hemoptysis/bloody sputum which were attributed to epistaxis, and he had lost about 50 pounds over the past year and a half which was attributed to increased physical activity. A chest CT was obtained and demonstrated innumerable patchy irregular pulmonary nodules throughout the mid and lower lungs bilaterally, which raised concern for post-infectious bronchiolitis obliterans or other infectious etiologies (Fig. 2A and B). An infectious workup was performed and was notable for elevated inflammatory markers and a sputum culture that grew Candida and *Staphylococcus aureus*. Flexible bronchoscopy revealed a tracheal mass 13 mm below the vocal cords that occluded 90% of the trachea (Fig. 3A). Debulking of the mass was performed by ENT via rigid bronchoscopy and histopathology confirmed low to intermediate-grade MEC and the presence of a mammalian mastermind like 2 (MAML2) gene rearrangement. Two months after his initial debulking procedure he underwent cricotracheal resection with tracheal anastomosis and margins showed no evidence of tumor (Fig. 3B). Repeat chest imaging 3 months after tumor debulking showed resolution of the previously noted bilateral pulmonary nodules (Fig. 2C and D). Repeat airway evaluation with ENT 13 months after his initial diagnosis showed no evidence of MEC on biopsy. His respiratory symptoms resolved and he no longer requires pulmonary follow up. He continues to be followed by ENT for surveillance airway evaluations.

### 2.2 Case 2

**Fig. 4:**
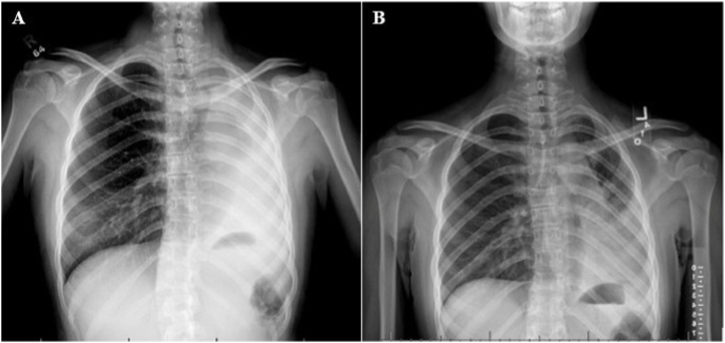
X-rays for case 2. A) Chest x-ray obtained at the time of presentation showed left hemithorax opacification with left-sided mediastinal shift. B) A spine x-ray to evaluate for scoliosis obtained one-year prior to her episode of large-volume hemoptysis showed airspace disease in the left lower lobe and lingula.

**Fig. 5:**
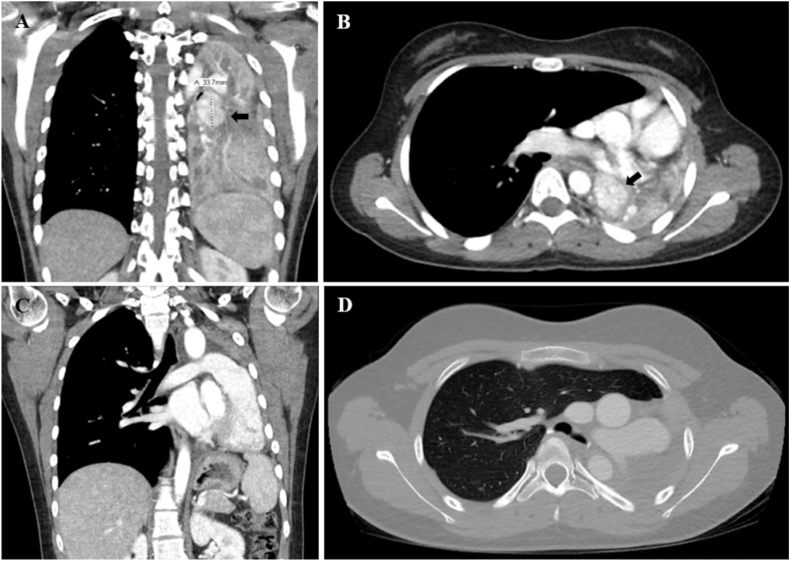
Chest CTs for case 2. A and B) Chest CT obtained prior to left pneumonectomy demonstrated a 3.4 cm × 2.4 cm x 3.3 cm enhancing round mass with complete left bronchus occlusion (black arrow), right lung hyperexpansion, and distal fluid-filled airways. C and D) Chest CT obtained 4 months after left pneumonectomy demonstrated right lung hypertrophy, left-sided shift of the heart and mediastinum, and no recurrence of the mass.

**Fig. 6:**
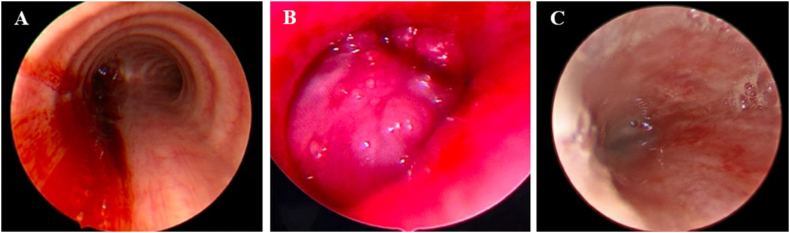
Bronchoscopy for case 2. A) Initial bronchoscopy showed frank blood and clots extending from the mid trachea to left mainstem bronchi. B) Initial bronchoscopy visualized an endobronchial mass in the left mainstem bronchus. C) Repeat bronchoscopy after endobronchial biopsies were obtained demonstrated persistent obstruction of the proximal left mainstem bronchus.

A previously healthy 14-year-old female presented with a one-year history of mild progressive dyspnea, exercise intolerance, and intermittent episodes of small-volume hemoptysis that the family had initially monitored at home. They sought medical care for her symptoms after she developed acute large-volume hemoptysis, which prompted hospital admission. Initial laboratory evaluation including blood gas, complete blood count, and coagulation panel was normal. Chest x-ray was notable for complete left lung atelectasis (Fig. 4A). Of note, a spine x-ray obtained one-year prior to evaluate for scoliosis showed mild collapse of the left lower lobe and lingula, though there was no further evaluation of these findings (Fig. 4B). Chest CT demonstrated an endobronchial mass with complete left mainstem bronchus occlusion, endobronchial extension, left pulmonary artery compression, and right lung hyperexpansion (Fig. 5A and B). Echocardiogram showed normal biventricular systolic function, despite complete distal left pulmonary artery compression. After multidisciplinary discussion, direct visualization of the mass was performed with flexible bronchoscopy and direct laryngobronchoscopy, confirming complete left bronchus occlusion with significant endobronchial extension after suctioning of frank blood and clots (Fig. 6A, B and C). Endobronchial biopsies were obtained with initial histopathology favoring bronchial adenoma over MEC, however, definitive diagnosis was challenging due to small biopsy sizes. Surgical exploration revealed extension of the endobronchial mass from the left lower lobe to the left upper lobe, necessitating complete left pneumonectomy. Subsequent histopathology of the left lung confirmed low-grade MEC without lymphovascular invasion. Genetic testing was positive for a MAML2 gene mutation. On follow-up the patient's symptoms had improved and a repeat chest CT showed mild right lung hypertrophy with slight mediastinal shift to the left hemithorax, without recurrence or metastasis (Fig. 5C and D).

### 2.3 Case 3

**Fig. 7:**
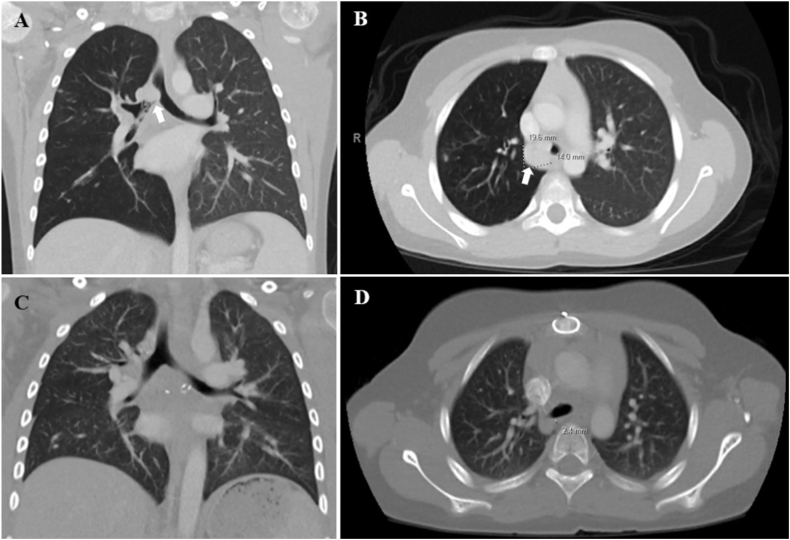
Chest CTs for case 3. A and B) Initial chest CT demonstrated a 2 cm × 1.4 cm x 2 cm lobulated mass invading the posterolateral right mainstem bronchus with near complete occlusion of the bronchus and invasion into the lower thoracic trachea (white arrow), with right lung hyperinflation, and diffuse scattered tree-in-bud opacities. C and D) Repeat chest CT 3 months after surgical resection of the right mainstem bronchus and re-anastomosis to the trachea demonstrated no persistent tumor or metastasis.

**Fig. 8:**
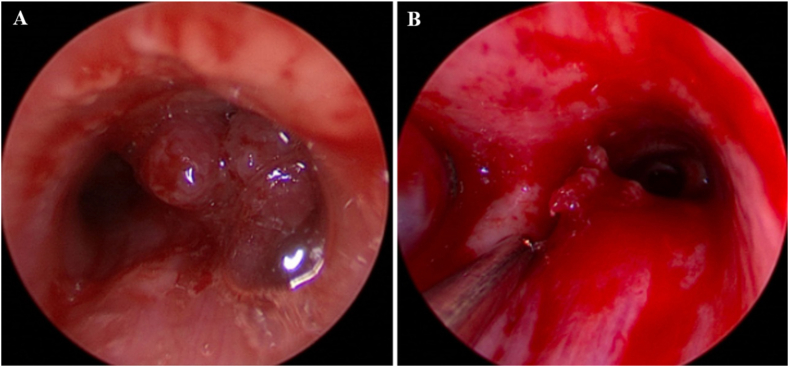
Bronchoscopy for case 3. A) Initial bronchoscopy demonstrated a near occlusive mass in the trachea and right mainstem bronchus. B) Repeat bronchoscopy after debulking procedure demonstrated a patent right mainstem bronchus.

A 7-year-old male previously diagnosed with moderate-persistent asthma presented with acute onset of hemoptysis. He had been treated with ICS-LABA, short courses of steroids, and antibiotics without improvement in his symptoms for the past five months. A prior chest x-ray showed mild hyperaeration of bilateral lungs that was initially attributed to asthma. After he presented with hemoptysis, further evaluation with a chest CT demonstrated a right tracheobronchial tumor with near complete occlusion and extension into the lower thoracic trachea (Fig. 7A and B). After a multidisciplinary evaluation, the patient underwent an endobronchial biopsy and debulking procedure (Fig. 8A and B). His histopathology findings were consistent with low-grade MEC, characterized by lymphovascular invasion, and a cyclic AMP-responsive element binding protein (CREB)-regulated transcription coactivator 3 (CRTC3)-MAML2 fusion gene mutation. This fusion is considered a key oncogenic driver and is associated with low-to intermediate-grade tumors, as well as a more favorable prognosis. The patient was initially discharged home with plans for further outpatient evaluation and treatment. Given the size and location of the tumor, proceeding directly with surgical resection would have required a pneumonectomy. Instead, the patient received a 7-week course of neoadjuvant chemotherapy with erlotinib (a tyrosine kinase inhibitor that works by blocking the epidermal growth factor receptor (EGFR)) to shrink the tumor and allow for less invasive surgical resection. The treatment was discontinued early due to development of progressive rash, pruritus, and pain. Repeat chest CT demonstrated a 20% decrease in mass size. The patient ultimately had a surgical resection of the right mainstem bronchus and re-anastomosis to the trachea. Post-operatively, the patient had normal pulmonary function testing, no evidence of persistent tumor or metastasis on repeat chest CT 3 months after surgical resection (Fig. 7C and D), and complete resolution of his symptoms.

### 2.4 Case 4

**Fig. 9:**
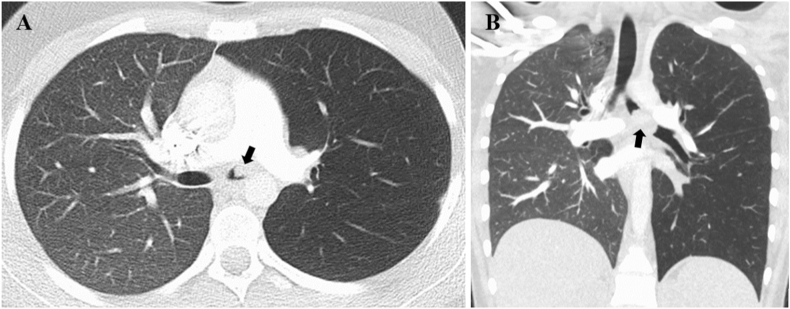
Chest CT for case 4. A) Axial and B) Coronal images demonstrated a 2.5 cm occlusive lesion within the left mainstem bronchus (black arrow) with resultant air trapping on the left side.

**Fig. 10:**
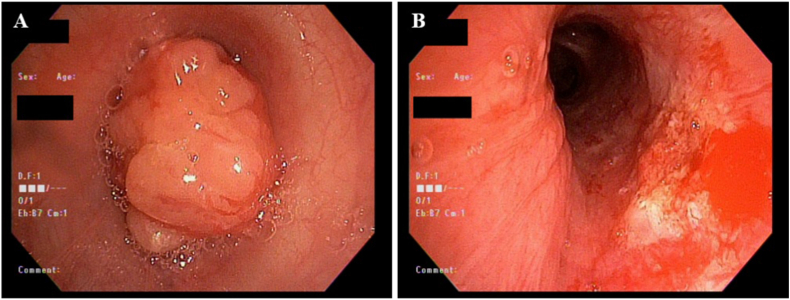
Bronchoscopies for case 4. A) Initial bronchoscopy demonstrated an endobronchial mass occluding the left mainstem bronchus. B) Left mainstem bronchus after removal of the endobronchial mass.

A 15-year-old female with a history of asthma developed gradually worsening shortness of breath, cough, wheezing, and mild hemoptysis which prompted presentation to the emergency room (ER) where she was treated for an asthma exacerbation with steroids and antibiotics. She had two subsequent presentations to the ER during which she was treated for presumed pneumonia. Due to persistent symptoms and increased hemoptysis a chest CT was obtained and revealed an obstructing left mainstem bronchus lesion measuring 2.5 cm in length with resultant air trapping (Fig. 9A and B). The lesion was visualized on bronchoscopy (Fig. 10A) and then removed by interventional pulmonology using an electrocautery snare and cryotherapy (Fig. 10B). The patient had nearly instantaneous relief of her symptoms. Histopathology revealed a low-grade MEC; MAML2 gene rearrangements were not tested for. She was referred to an outside facility with pediatric specialty care where she underwent slide bronchoplasty to ensure clear margins. Pathologic analysis of hilar lymph nodes and the excised portion of the LMSB bronchus during the slide bronchoplasty were negative for malignancy. She remains well on follow-up 7 years later.

## 3 Discussion

### 3.1 Clinical presentation

Our four cases highlight the wide variety of clinical presentations associated with tracheobronchial MEC, ranging from a very indolent presentation occurring for a year to acute large volume hemoptysis (Table 1). Our patients’ symptoms included cough, dyspnea, weight loss, hemoptysis, wheezing, and pneumonia, which are consistent with previously published studies [1,4,6,7,[9], [10], [11], [12]]. Unfortunately, these symptoms are not specific to tracheobronchial MEC and are often attributed to other causes, which contributes to a delay in diagnosis (prior literature has reported delays of 2-30 months) [[13], [14], [15], [16], [17]].

**Table 1:** Summary of patient cases.

Patient	Age	Sex	Symptoms	Spirometry	Location	Histopathology	Management	Outcome
Case 1	14 years old	M	One year of cough, wheezing, exercise intolerance with progression to hemoptysis/bloody sputum, weight loss	Flattening of inspiratory and expiratory loops	Tracheal mass, below vocal cords	Low to intermediate-grade MEC, MAML2 gene rearrangement	Initial flexible and rigid bronchoscopy with mass debulking. Ultimately required cricotracheal resection and tracheal anastomosis.	Disease free 2 years after surgery
Case 2	14 years old	F	One year of dyspnea, exercise intolerance, intermittent small-volume hemoptysis with progression to acute large volume hemoptysis	Not performed	Endobronchial mass with extension from left lower lobe to left upper lobe	Low-grade MEC, MAML2 gene mutation	Initial flexible bronchoscopy with endobronchial biopsy. Ultimately required complete left pneumonectomy.	Disease free 3 years after surgery
Case 3	7 years old	M	5 months of cough, wheezing with progression to acute hemoptysis	Not performed	Tracheobronchial tumor with near complete occlusion and extension into lower thoracic trachea	Low-grade MEC, CRTC3-MAML2 fusion gene mutation	Neoadjuvant chemotherapy with Erlotinib x7 weeks. Ultimately required surgical resection of right mainstem bronchus and tracheal re-anastomosis.	Disease free 3.5 years after surgery
Case 4	15 years old	F	Indolent dyspnea, cough, wheezing, and mild hemoptysis with progression to increased hemoptysis	Not performed	Endobronchial mass in left mainstem bronchus	Low-grade MEC, MAML2 gene not tested	Flexible bronchoscopy with mass removal by electrocautery snare and cryotherapy. Later required slide bronchoplasty.	Disease free 7 years after surgery

Three of our patients were previously treated for asthma without resolution of their symptoms before further workup revealed the presence of lesions in the large airways. Case 1 was particularly concerning for its indolent and non-specific presentation. He did not have the large volume hemoptysis or acute presentation that was the hallmark of our other cases. He had persistent flattening of his inspiratory and expiratory loops, which should raise concern for a fixed airway obstruction (Fig. 1); however, this was not identified until after the tracheal mass was seen on bronchoscopy. Reports of suboptimal effort on the first two studies (Fig. 1A and B) may have impaired the identification of the flattened loops. Although the truncated loops were not initially identified, additional workup for his persistent symptoms lead to a definitive diagnosis. No spirometry testing was performed prior to debulking procedures or tumor excision for the other three patients.

Additionally, three patients had prior episodes of hemoptysis that were misattributed to epistaxis, asthma, or pneumonia, and one patient's significant weight loss was confounded by this patient's claim of increased physical activity. Jaramillo et al., ’s 2016 review of 145 cases of tracheobronchial MEC found that 20% of patients had hemoptysis and 5% of patients had weight loss [4]. Our cases emphasize the importance of having a broad differential including rare diseases such as a primary lung neoplasm when a patient presents with hemoptysis (especially recurrent hemoptysis) and/or weight loss in the setting of other pulmonary symptoms.

The initial chest CTs for three of our cases demonstrated findings consistent with bronchial obstruction (Fig. 5, Fig. 7, Fig. 9). We suspect that the pulmonary nodules seen on the initial chest CT for case 1 were due to mucus impaction, which resolved after surgical removal of the tracheal mass (Fig. 2). Unfortunately, the initial chest CT did not include the subglottic space and therefore did not visualize the tracheal mass, which contributed to a delay in diagnosis. Case 2 also had a delay in diagnosis despite abnormal pulmonary findings on the spine x-ray obtained prior to her presentation with large-volume hemoptysis (Fig. 4B).

### 3.2 Diagnosis

In order to make a diagnosis of tracheobronchial MEC, histopathological examination of a tissue biopsy is required because other airway lesions may have a similar presentation. The differential for a tracheobronchial lesion includes infection (e.g. tuberculosis, nontuberculous mycobacteria, fungus), benign lesions (e.g. hemangioma, adenoma, hamartoma), and malignant tumors (e.g. carcinoid tumors, inflammatory myofibroblastic tumors, rhabdomyosarcoma). Tissue biopsies can be obtained via interventional pulmonology techniques such as cryobiopsy and endobronchial forceps. The invention of the 1.1 mm cryoprobe has increased utilization of cryobiopy in children because it can fit through the working channel of pediatric bronchoscopes. A multicenter study on airway cryotherapy in children by Schramm et al. found that 5 of the 6 endobronchial cryobiopsies yielded a successful histopathological evaluation. Complications associated with cryobiopsy include superficial mucosal bleeding (most commonly), bleeding requiring local application of vasoactive substances, pneumothorax, and bronchospasm [19].

The World Health Organization classifies tracheobronchial MEC as an “epithelial tumor characterized by the presence of squamoid cells, mucin-secreting cells and cells of intermediate type.” Low-grade tracheobronchial MEC predominantly has cystic changes and is made up of mucin-secreting cells, whereas high-grade tracheobronchial MEC is more solid and is made up of mostly squamoid cells and intermediate cells [8]. There are several different grading systems utilized, with the most common being the Armed Forces Institute of Pathology (AFIP) system [20], the Brandwein system [21], and the modified Healey system [22].

Prior studies have demonstrated that rearrangements of the MAML2 gene are observed in tracheobronchial MEC and that the presence of this rearrangement can be used to distinguish tracheobronchial MEC from other types of carcinoma [[23], [24], [25], [26], [27]]. MAML2 is a Notch receptor coactivator and prior studies have demonstrated that MAML2 gene rearrangements result in altered Notch and CREB signaling [[28], [29], [30], [31]]. Notch signaling is involved in cell-cell communication and regulates cell differentiation, and CREB is a cellular transcription factor. MAML2 rearrangements have been identified in low-, intermediate- and high-grade tracheobronchial MEC, though some studies suggest that this gene rearrangement is more commonly seen in low- and intermediate-grade tracheobronchial MEC [23,24]. The prognostic significance of the MAML2 rearrangement is unclear at this time.

### 3.3 Management

Given the rarity of tracheobronchial MEC, treatment is determined on a case-by-case basis, but surgical resection is the mainstay of therapy because tracheobronchial MEC rarely metastasizes and surgical resection is more likely to be curative compared to endoscopic resection (recurrence can occur if the entire tumor is not removed) [4,12,[32], [33], [34]]. Prior studies have found that lobectomy, sleeve resection (including segmental tracheal and carinal resections), and pneumonectomy were the most common surgical approaches. Less common surgical approaches include endoscopic resection, non-endoscopic local resection, and segmentectomy [1,4,33]. All four of our patients underwent surgical resection, though case 3 was treated with neoadjuvant chemotherapy with erlotinib prior to surgical resection. The use of adjuvant chemotherapy and/or radiation has been reported in cases of recurrence after surgical resection or when complete surgical resection is not possible [1,4,35,36].

While some MECs exhibit EGFR overexpression or activating mutations, evidence supporting the use of erlotinib in pediatric tracheobronchial MEC is minimal. A few case reports and small series in adults with pulmonary MEC have described partial responses to EGFR inhibitors, particularly in high-grade, unresectable, or metastatic tumors with confirmed EGFR alterations [[37], [38]]. In pediatric patients, MEC is generally low-grade and amenable to surgery, making systemic, targeted therapy such as erlotinib rarely indicated. However, in rare cases where complete resection is not anatomically possible, difficult, or in high-grade, recurrent, or metastatic disease, erlotinib may be considered as an individualized treatment option.

Although interventional pulmonology is more commonly used in the adult population, its use in children is growing and we may see interventional pulmonology techniques utilized in the diagnosis and management of tracheobronchial MEC more frequently in the future. One of our patients (case 4) was initially treated with electrocautery snare and cryotherapy, and then underwent slide bronchoplasty. In 2015, Wang et al. published a case series that highlighted the use of endoscopic resection via argon plasma coagulation and carbon dioxide cryotherapy in 6 children with low-grade tracheobronchial MEC. Successful endoscopic resection without disease recurrence (with follow-up ranging between 16 and 72 months) was achieved in 5 of the 6 children. The sixth child underwent endoscopic resection but due to failure to recanalize the lumen, they ultimately required a left upper lobectomy [39]. Another study published by Wong et al., in 2023 reported the use of laser ablation via rigid bronchoscope for low-grade tracheobronchial MEC in 3 children. All 3 children were disease-free at follow-up ranging from 3 to 6 years [40]. It's important to note that both of these studies only included patients with low-grade tracheobronchial MEC, which typically does not metastasize. The use of endoscopic resection would not be the best approach for treating high-grade tracheobronchial MEC which can invade the pulmonary parenchyma, involve regional lymph nodes, and can metastasize to other organs [8]. Further studies are needed to assess the safety, efficacy, and indications of interventional procedures (such as transbronchial and endobronchial biopsy, transbronchial needle aspiration with endobronchial ultrasound, cryotherapy, laser-assisted procedures, and airway stents) in children.

### 3.4 Outcomes

Tracheobronchial MEC typically has a favorable outcome after surgical resection. However, patients with high-grade tracheobronchial MEC have worse outcomes because it can invade the pulmonary parenchyma, involve regional lymph nodes, and can metastasize to other organs [4,8]. Low-grade tracheobronchial MEC typically does not metastasize (less than 5% of cases spread to regional lymph nodes). Jaramillo et al.’s review found that of the 120 patients with follow up, there was an overall disease-free survival of 96% (follow-up ranged from 1 month to 23 years, mean: 4.2 years, and median: 2.5 years) [4]. There were 5 deaths (3 patients had high-grade tracheobronchial MEC, the grade was unknown for the other 2 patients) and two patients died despite additional surgical intervention, chemotherapy, and/or radiation [4,35,36].

All four of our patients had low-grade or low to intermediate-grade tracheobronchial MEC and are disease free at the time of this publication (follow up post-surgical resection ranges from 2 to 7 years).

## 4 Conclusion

Tracheobronchial MEC is a rare malignant primary lung neoplasm which presents with non-specific symptoms and requires a high index of suspicion for timely diagnosis. Symptoms such as hemoptysis and unexplained weight loss in patients with refractory respiratory symptoms should prompt consideration for cross-sectional imaging to assess for a pulmonary mass. Diagnosis is made via histopathologic examination of a tissue sample. Treatment ranges from simple endobronchial excision to invasive surgical excision, and may include adjuvant chemotherapy and/or radiation; outcomes are generally favorable. Our case series highlights the importance of astute observation given the diversity in presentations and a multidisciplinary approach to the management of tracheobronchial MEC. As the use of pediatric interventional pulmonology grows, further studies are needed to elucidate the role of interventional pulmonology techniques such as cryotherapy, laser ablation, and endoscopic resection in the diagnosis and management of tracheobronchial MEC. These techniques will hopefully decrease the need for invasive surgical excision in low-grade tracheobronchial MEC.

## CRediT authorship contribution statement

**Kalen Hendra:** Conceptualization, Visualization, Writing – original draft. **Muhammad Ijlal Khan:** Writing – original draft. **Mitali Thanawala:** Writing – original draft. **Manuel Silva-Carmona:** Writing – review & editing. **Eric D. Zee:** Conceptualization, Supervision, Writing – review & editing.

## Funding sources

This research did not receive any specific grant from funding agencies in the public, commercial, or not-for-profit sectors.

## Declaration of competing interest

The authors declare that they have no known competing financial interests or personal relationships that could have appeared to influence the work reported in this paper.
